# Risk factors affecting the feeding site predilection of ticks on cattle in Ghana

**DOI:** 10.1007/s10493-024-00906-7

**Published:** 2024-04-05

**Authors:** Seth Offei Addo, Ronald Essah Bentil, Mba-tihssommah Mosore, Eric Behene, Julian Adinkrah, Janice Tagoe, Clara Yeboah, Bernice Olivia Ama Baako, Dorcas Atibila, Sandra Abankwa Kwarteng, Kwaku Poku-Asante, Ellis Owusu-Darbo, Victor Asoala, Daniel Lartei Mingle, Edward Owusu Nyarko, Anne T. Fox, Andrew G. Letizia, Joseph Diclaro William, Shirley Nimo-Paintsil, James F. Harwood, Samuel Kweku Dadzie

**Affiliations:** 1 Parasitology Department, Noguchi Memorial Institute for Medical Research, University of Ghana, Legon, Accra, Ghana; 2 U.S. Naval Medical Research Unit EURAFCENT, Accra, Ghana; 3 Navrongo Health Research Centre, Navrongo, Upper East Region, Ghana; 4 Entomology Unit, Department of Clinical Laboratory, Kintampo Health Research Centre, Kintampo, Ghana; 5 Department of Theoretical and Applied Biology, Kwame Nkrumah University of Science and Technology, Kumasi, Ghana; 6 School of Public Health, Kwame Nkrumah University of Science and Technology, Kumasi, Ghana; 7 Public Health Division, 37 Military Hospital, Ghana Armed Forces Medical Service, Accra, Ghana; 8 Infectious Diseases Directorate, Naval Medical Research Center, Silver Spring, MD USA; 9 Navy Entomology Center for Excellence, Jacksonville, FL USA; 10 U.S Naval Medical Research Unit EURAFCENT, Sigonella, Italy

**Keywords:** Ixodid ticks, Cattle, Predilection sites, Ecological zones, Ghana

## Abstract

Ticks are important disease vectors affecting animal health and causing substantial economic loss, especially in the tropics and subtropics. To examine the tick burden of cattle and associated risk factors for tick infestation, ticks were collected from 388 cattle within five regions in Ghana. Most of the cattle were males (50.3%) and generally older than 3 years (65%). Of the animals sampled, 2187 ticks were collected with a mean tick burden of 5.6 ticks per cattle, and the average tick burden on the udder/scrotum being significantly higher than in the anal region (Generalized Linear Mix Model [GLMM], *p* = 0.01197). The tick species identified were predominantly *Amblyomma variegatum* (42.6%) and *Hyalomma rufipes* (26.2%). High proportions of cattle examined were found to have *A. variegatum* infesting the udder/scrotum. Furthermore, *H. rufipes* infested mostly the anal region compared to other examined body parts (OR 14.8, 95% CI 8.6–25.4, *p* < 0.001). Using the GLMM, tick abundance was found to be significantly higher in cattle older than 3 years. The tick burden in the udder/scrotum was higher than that from the chest and leg/thigh of the cattle (GLMM, *p* < 0.05). The tick burden at the anal region was also significantly higher than the leg/thigh and chest. This study indicates that the preferred attachment sites of ticks on cattle are species-dependent and effective treatment with acaricides should take into consideration the udder/scrotum and anal regions as well as prioritizing older cattle.

**Supplementary Information:**

The online version contains supplementary material available at 10.1007/s10493-024-00906-7.

## Introduction

In the tropical and subtropical regions, ticks are vectors for multiple pathogens that cause diseases in animals and humans (Balinandi et al. 2020; Estrada-Peña and De La Fuente 2014). With the increasing trade of livestock among African countries (Chand 2020) and the extensive transboundary migrations either in search of pasture or due to conflicts (Zannou et al. 2021), ticks can more readily infiltrate new regions and further spread tick-borne pathogens. Tick-borne disease negatively affects animal health and associated economic activity resulting in significant financial loss (Rajput et al. 2006; Vesco et al. 2011), with nearly 80% of livestock mortality caused by tick-borne diseases (Cleaveland et al. 2001; Cumming 2002). Multiple tick-borne pathogens can cause acute and chronic morbidity in humans, which are threats to public health (Rajput et al. 2006; Sarani et al. 2014). Despite this impact on human and animal health, control efforts have proven ineffective due to a lack of knowledge on the ecology of ticks and their resting preferences as well as human, biological and environmental factors that affect their biology (Cleaveland et al. 2001; Cumming 2002). To better understand the ecology of ticks, there is a need to investigate how demographic factors such as age, sex and the ecological zones they live in affect tick infestation on cattle (Cumming 1999; Dantas-Torres 2008).

Ticks prefer areas on an animal's body where the skin is thin and blood flow plentiful, like the inguinal region and external genital area (Hurtado and Giraldo-Ríos 2018). This can be observed in the case of *A. variegatum* which has a preference for the udder of livestock leading to drastically reduced milk production, serious wounds (Stachurski 2000) and impaired growth (Pegram and Oosterwijk 1990).

Within Africa, about 50 tick species have been documented to infest domestic animals (Walker et al. 2003) with the genera *Rhipicephalus*, *Hyalomma* and *Amblyomma* having the greatest impact on livestock and human health (Reye et al. 2012; Balinandi et al. 2020). In Ghana, tick species of the genera *Amblyomma*, *Hyalomma* and *Rhipicephalus* have been identified to infest livestock (Ntiamoa-Baidu et al. 2004). Additionally, tick-borne pathogens including Crimean-Congo Haemorrhagic Fever Virus (CCHFV), Dugbe Virus and *Rickettsia africae* have been reported in tick species (Akuffo et al. 2016; Kobayashi et al. 2017; Addo et al. 2023a). However, there is limited data on the ecology of ticks and how this affects tick infestation in the country. Given the veterinary and economic importance of ticks, there is a need to understand their biology, especially concerning their feeding and resting site preferences on cattle. This will provide essential information to develop effective control methods, given the apparent threat of zoonotic pathogen transmission in Ghana due to the dependence on livestock production. In this study, the burden of tick infestation and their feeding and resting preferences on cattle from different ecological zones in Ghana were investigated.

## Methods

### Study sites

**Fig. 1:**
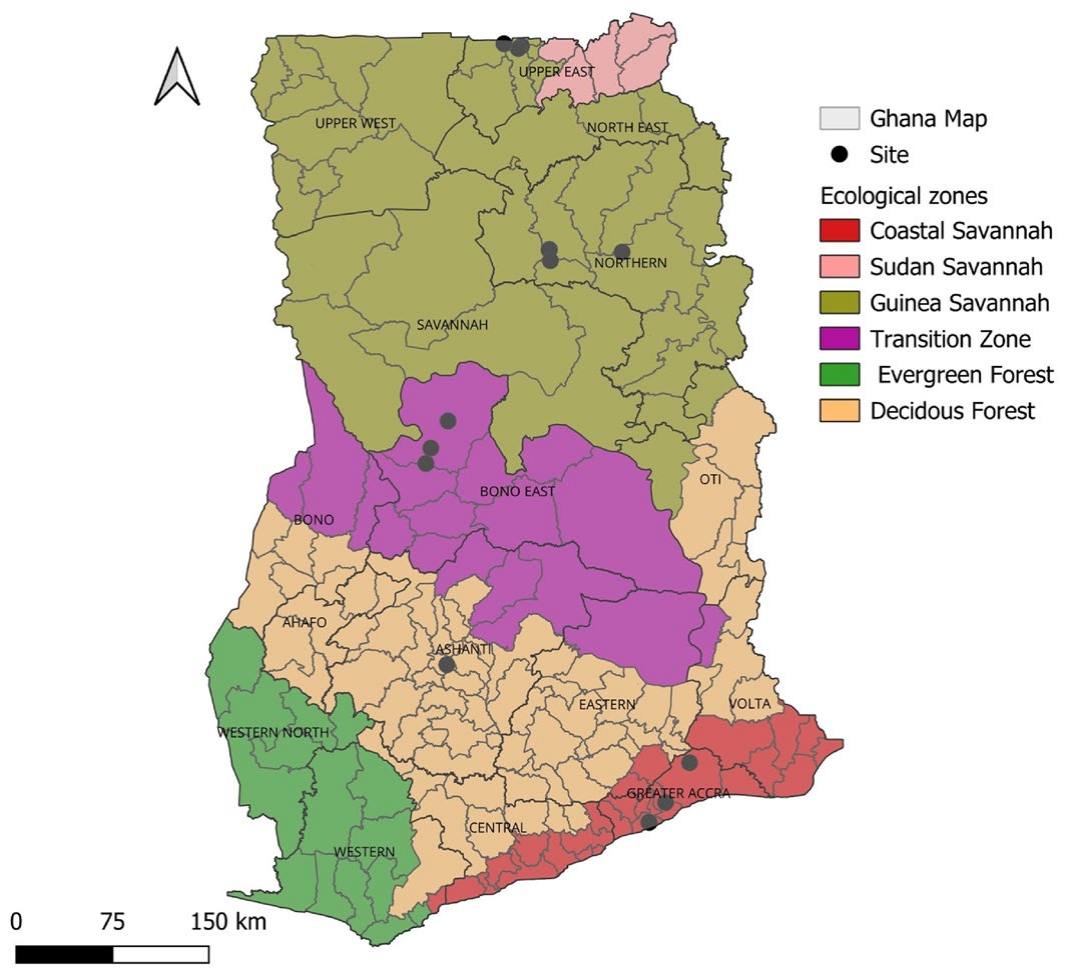
A Regional map of Ghana indicating sampling sites for ticks within the different ecological zones

Ticks were collected from five different sampling locations in Ghana namely, Upper East (Abattoir, Cattle Market, Nakong), Northern (Airforce Base, Airborne Force, Kamina, Daboya), Bono East (Sunuase, Dawadawa, Cattle Market, Abattoir), Ashanti (Kumasi-abattoir) and Greater Accra (3MTD-Burma Camp, 1BN-Michel Camp, Asutsuare) (Fig. 1). The sampling took place from January to August 2020.

## Tick collection and Identification

Using Epi Info version 6, a required sample size of 388 livestock (rounded up from the calculated number of 374) was calculated. Sample size calculations were made with the following assumptions: population size (estimated number of livestock in the study areas based upon local veterinarian estimate) of 16,633 livestock; anticipated prevalence rate of 45%; and a 5% confidence level. Eighty livestock were selected from each of the five regions and with the help of the herdsmen, livestock infested with ticks were identified and sampled. All body parts of the livestock were examined and ticks identified were collected using blunt forceps and placed in labelled vials containing RNA later (Qiagen, Germany). Ticks obtained from the same region of the animal were put into the same vial. In the laboratory, ticks were morphologically identified with the aid of a dissecting microscope using taxonomic keys (Walker et al. 2003) and individually stored at − 80 °C in 2 ml Eppendorf vials containing RNA Later. The taxonomic keys used in the morphological identification is an illustrated guide that outlines a straightforward three-step method for recognizing domestic animal ticks in Africa.

Generally, the mouthparts of the hard ticks identified in this study project forward, they have a scutum and often have eyes that are visible dorsally. Ticks of the genera *Amblyomma* and *Hyalomma* are bigger with large eyes, long mouthparts projecting to the anterior part of the body and pale rings on segments of their legs. With the genus *Rhipicephalus*, the ticks are medium in size with short mouthparts, eyes present and coxae 1 having large and equal paired spurs. *Boophilus* ticks are smaller in size, have short mouthparts, the eyes are small or absent and the coxae 1 has small paired spurs or single spur. To further identify ticks at the species level, the taxonomic keys provide pictorial unique features. For example, *A. variegatum* has small to medium punctuations, the posterior lips of the female genital aperture is U shaped and the males have enamel patterns often without lateral spots and no enamel on the festoons.

Animal data such as sex and age were obtained from the animal handler and recorded. Animals less than or equal to 3 years old were considered young while those 4 years or more were considered old.

### Molecular identification of tick species

Ticks that were engorged and difficult to morphologically identify to the species level were subject to molecular identification. Total nucleic acid was extracted from each tick using the QIAamp Mini Kit (Qiagen, Valencia, CA, USA) (Crowder et al. 2010). To determine the tick species, primers TickCO1-F (TACTCTACTAATCATAAAGACATTGG) and TickCO1-R (CCTCCTCCTGAAGGGTCAAAAAATGA) that amplify the 660-bp segment of the mitochondrial COI gene were used (Barrett and Hebert 2005).

A total of 50 μl was used for each PCR reaction, which included 25 μl of GoTaq® Green Master Mix (2x), 1 μM of both forward and reverse primers, 18 μl of nuclease-free water and 5 μl of DNA as template. Each PCR reaction contained both positive (tick isolate) and negative (nuclease-free water) controls. Mastercycler X50-PCR Thermocycler (Eppendorf, Germany) was used to run the PCR. The cycling conditions were as follows: a first hold at 95 °C for 5 min; a second hold at 95 °C for 30 s, 48 °C for 30 s and 72 °C for 1 min at 34 cycles; and a third hold at 72 °C for 5 min. At 4 °C, the reaction was maintained. Using 2 g of agarose in 100 ml of 1 × Tris Acetate EDTA, a 2% agarose gel was made. It was then stained with 5 μl of SYBR® Safe DNA gel stain. Each PCR product (5 μl) was placed onto the gel along with a 100 bp ladder from New England BioLabs and run for 30 min at 100 V. Afterwards, the gel was examined using a Molecular Imager® Gel Doc (Bio-Rad).

For Sanger sequencing, the PCR products were sent to Macrogen Europe B.V. in Amsterdam, Netherlands. Using Chromas (version 2.6.6), each sequence obtained in this study was viewed after which MEGA (version 10.0.5) was used to edit, clean and generate a consensus sequence. The consensus sequences were subsequently compared to various sequences that had been submitted to the NCBI database (https://blast.ncbi.nlm.nih.gov/Blast.cgi).

## Data analysis

Statistical analysis was done using R version 4.1.0. Descriptive statistics were used to compare animal and tick characteristics. Categorical variables were described using percentages and frequencies. Count variables were described using mean and standard deviations. Kruskal Wallis or Mann Whitney testing was used to determine the association between animal demographic characteristics and the count of ticks from different body parts of livestock. To determine the association between tick species and the animal’s age, sex and region from where it was sampled, each tick species was treated as a dichotomous variable (dependent) and a univariate logistic regression was fitted with age, sex and the region sampled as an independent variable. To identify the risk factors of tick burden on the livestock, a negative binomial generalized linear mixed model (GLMM) was used. Statistical significance was set at *p* < 0.05.

## Results

### Demographic characteristics of cattle sampled

A total of 388 cattle were examined from the study sites (10 farms, 3 abattoirs and 2 cattle markets). Out of the total number of cattle examined, approximately 50% were females while 65.2% were greater than 3 years old (Table 1, S1Table). The total number of ticks obtained from the cattle were 2187 with a majority (35.1%) obtained from the Ashanti region of Ghana. Approximately 54% of the ticks were obtained from the udder/scrotum of the cattle with the average number of ticks collected being 3.0 (SE = 0.27) (Table 2, S2 Table).

**Table 1:** Prevalence and mean distribution of ticks sampled from cattle

	Number of Animals sampled	Number of ticks collected	Percentage Tick Abundance	Average number of ticks collected
	*N* (%)		(%)	Mean (SD)
*Sex of animal*				
Male	195 (50.3)	1224	56.0	6.3 (6.7)
Female	193 (49.7)	963	44.0	5.0 (3.6)
*Age (years)*				
≤ 3	136 (35.0)	558	25.5	4.1 (3.6)
> 3	252 (65.0)	1629	74.5	6.5 (6.0)
*Ecological zone*				
Coastal Savannah	69 (17.8)	394	18.0	5.7 (4.3)
Deciduous Forest	80 (20.6)	768	35.1	9.6 (8.1)
Transition Zone	80 (20.6)	407	18.6	5.1 (4.4)
Guinea Savannah	159 (41.0)	618	28.3	3.9 (3.2)
Total	388	2187	100	5.6 (5.4)

**Table 2:** Occurrence of tick species sampled from the various body parts of cattle

	Udder/scrotum	Abdomen	Chest	Anal region	Head/neck	Leg/Thigh	Total	Percentage (%)
*A. variegatum*	691	74	74	85	4	10	938	42.9
*H. rufipes*	170	20	9	370	4	2	575	26.3
*H. truncatum*	176	23	25	116	2	3	345	15.8
*H. dromedarii*	1	0	1	8	0	1	11	0.5
*H. marginatum*	0	0	0	2	0	0	2	0.1
*R. annulatus*	16	0	4	0	1	0	21	1.0
*R. decoloratus*	15	0	13	1	1	1	31	1.4
*R. evertsi evertsi*	4	0	3	5	0	4	16	0.7
*R. geigyi*	9	7	14	0	0	9	39	1.8
*R. microplus*	88	9	40	63	1	4	205	9.4
*R. turanicus*	1	0	0	3	0	0	4	0.2
Total	1171	133	183	653	13	34	2187	
Percentage (%)	53.5	6.1	8.4	29.9	0.6	1.6		

## Tick species composition and area where they were collected

**Fig. 2:**
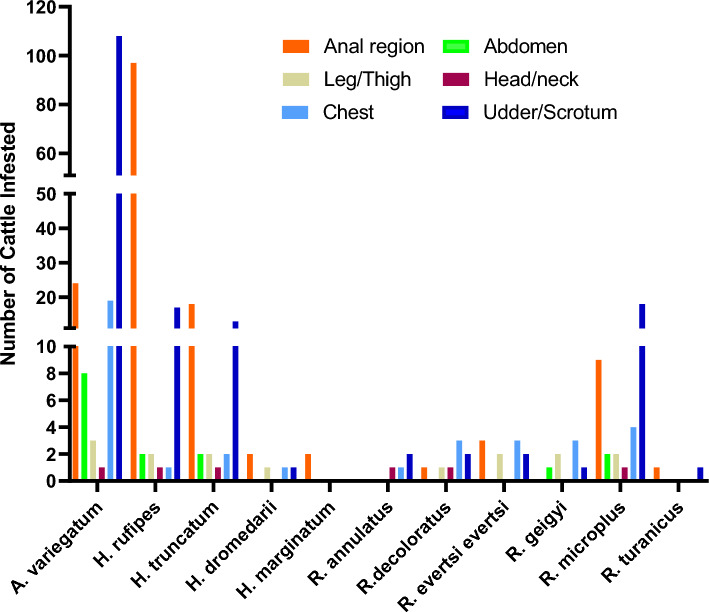
The preferred site of attachment of tick species on Cattle (*n* = 388)

Of the total number of ticks collected, 42.9% were *A. variegatum* and 26.3% were *H. rufipes*. It was observed that more male cattle were infested with *A. variegatum* than females (49.7% compared to 34.2%; OR 1.9, 95% CI 1.3–2.9, *p* = 0.002) (Table 3). *Amblyomma variegatum* infestation did not significantly differ among the younger and older cattle (OR 1.0, 95% CI 0.7–1.6, *p* = 0.977). Also, high proportions of cattle examined were found to have *A. variegatum* attached to the udder/scrotum (Fig. 2). Cattle from the Deciduous Forest had a higher likelihood of *A. variegatum* infestation as compared to those from the other ecological zones (OR 3.8, 95% CI 2.3–6.5, *p* < 0.001). The male cattle were less likely to have *H. rufipes* infestation as compared to the females (OR 0.5, 95% CI 04–0.8, *p* = 0.007). Regarding the preferred site of attachment, anal regions were more likely to be infested with *H. rufipes* as compared to the other body parts. Cattle in the Deciduous Forest ecological zone were also more likely to be infested with *H. rufipes* than those in the other ecological zones (OR 0.3, 95% CI 0.2–0.6, *p* = 0.001). Cattle from the Transition zone were more likely to be infested with *H. truncatum* (OR 0.2, 95% CI 0.0–0.9, *p* = 0.032) compared to cattle from the Coastal savannah which are more likely to be infested with *Rhipicephalus microplus* (OR 2.3, 95% CI 1.1–5.0, *p* = 0.031) (S3Table; Table 4).

**Table 3:** Tick species associated with cattle sex

Tick species	Sex of cattle	No. of animals examined	No. of animals infested *n* (%)	OR (95% CI)	*p*-value
*A. variegatum*	Female	193	66 (34.2)	1 (ref)	
	Male	195	97 (49.7)	1.9 (1.3–2.9)	0.002*
*H. rufipes*	Female	193	72 (37.3)	1 (ref)	
	Male	195	48 (24.6)	0.5 (0.4–0.8)	0.007*
*H. truncatum*	Female	193	18 (9.3)	1 (ref)	
	Male	195	18 (9.2)	1.0 (0.5–2.0)	0.974
*H. dromedarii*	Female	193	2 (1.0)	1 (ref)	
	Male	195	0 (0.0)	1	–
*H. marginatum*	Female	193	1 (0.5)	1 (ref)	
	Male	195	1 (0.5)	0.99 (0.06–15.9)	0.994
*R. annulatus*	Female	193	1 (0.5)	1 (ref)	
	Male	195	1 (0.5)	0.99 (0.06–15.9)	0.994
*R. decoloratus*	Female	193	3 (1.6)	1 (ref)	
	Male	195	3 (1.5)	0.99 (0.2–4.96)	0.990
*R. evertsi evertsi*	Female	193	5 (2.6)	1 (ref)	
	Male	195	5 (2.6)	0.99 (0.3–3.5)	0.987
*R. geigyi*	Female	193	3 (1.6)	1 (ref)	
	Male	195	4 (2.1)	1.3 (0.3–6.0)	0.714
*R. microplus*	Female	193	21 (10.9)	1 (ref)	
	Male	195	14 (7.2)	0.6 (0.3–1.3)	0.206
*R. turanicus*	Female	193	1 (0.5)	1 (ref)	
	Male	195	1 (0.5)	0.99 (0.06–15.9)	0.994

*Statistically significant; OR and *p*-value were obtained using a univariate logistic regression

OR is defined as odds ratio

**Table 4:** Tick species associated with cattle age

Tick species	Sex of cattle	No. of animals examined	No. of animals infested *n* (%)	OR (95% CI)	*p*-value
*A. variegatum*	≤ 3 years	136	57 (41.9)	1 (ref)	
	> 3 years	252	106 (42.1)	1.0 (0.7–1.6)	0.977
*H. rufipes*	≤ 3 years	136	38 (27.9)	1 (ref)	
	> 3 years	252	82 (32.5)	1.2 (0.8–2.0)	0.350
*H. truncatum*	≤ 3 years	136	11 (8.1)	1 (ref)	
	> 3 years	252	25 (10.0)	1.3 (0.6–2.6)	0.553
*H. dromedarii*	≤ 3 years	136	2 (1.5)	1 (ref)	
	> 3 years	252	0 (0.0)	1	–
*H. marginatum*	≤ 3 years	136	0 (0.0)	1 (ref)	
	> 3 years	252	2 (0.8)	–	–
*R. annulatus*	≤ 3 years	136	1 (0.7)	1 (ref)	
	> 3 years	252	1 (0.40)	0.5 (0.03–8.7)	0.662
*R.decoloratus*	≤ 3 years	136	2 (1.5)	1 (ref)	
	> 3 years	252	0 (0.0)	1	-
*R. evertsi evertsi*	≤ 3 years	136	4 (2.9)	1 (ref)	
	> 3 years	252	6 (2.4)	0.8 (0.2–2.9)	0.740
*R. geigyi*	≤ 3 years	136	3 (2.2)	1 (ref)	
	> 3 years	252	4 (1.6)	0.7 (0.2–3.2)	0.664
*R. microplus*	≤ 3 years	136	14 (10.3)	1 (ref)	
	> 3 years	252	21 (8.3)	0.8 (0.4–1.6)	0.521
*R. turanicus*	≤ 3 years	136	1 (0.7)	1 (ref)	
	> 3 years	252	1 (0.4)	0.5 (0.03–8.7)	0.662

*Statistically significant; OR and *p*-value were obtained using a univariate logistic regression

OR is defined as odds ratio

## Risk factors for tick infestation

In the GLMM, tick abundance was significantly higher in cattle > 3 years old compared to ≤ 3 years old (*p* < 0.001). Tick burden in male cattle was not significantly higher than the females (GLMM, *p* = 0.0744). Ticks were identified on the udder/scrotum of 43% of the cattle. The tick burden in the udder/scrotum was higher than that from the anal, chest, head/neck, abdomen and leg/thigh of the cattle (GLMM, *p* < 0.05). However, tick burden at the anal region was significantly higher than leg/thigh, head/neck, abdomen and chest. Tick burden from the abdomen was also significantly higher than the leg/thigh and head/neck (Table 5).

**Table 5:** Risk factor analysis of tick burden on cattle using generalized linear mixed model

	Estimate	SE	z	*p*-value
Intercept	− 1.48	0.22	− 6.86	< 0.001*
*Age of animal*				
> 3 years vs. ≤ 3 years	0.35	0.14	2.55	0.0109*
*Sex of animal*				
Male vs. Female	− 0.24	0.13	− 1.78	0.0744
*Animal body part*				
Anal vs. Abdomen	1.82	0.21	8.36	< 0.001
Leg/Thigh vs. Abdomen	− 1.28	0.26	− 4.84	< 0.001
Head/neck vs. Abdomen	− 1.80	0.29	− 6.12	< 0.001
Chest vs. Abdomen	0.43	0.21	− 2.05	0.30236
Udder/Scrotum vs. Abdomen	2.47	0.22	11.12	< 0.001*
Leg/Thigh vs. Anal	− 3.09	0.25	− 12.50	< 0.001*
Chest vs. Anal	− 1.38	0.19	− 7.11	< 0.001*
Head/neck vs. Anal	− 3.62	0.29	− 12.52	< 0.001
Udder/Scrotum vs. Anal	− 0.65	0.17	3.73	0.00261
Chest vs. Leg/Thigh	1.71	0.25	6.77	< 0.001
Head/neck vs. Leg/Thigh	− 0.52	0.33	− 1.60	0.59109
Udder/Scrotum vs. Leg/Thigh	3.75	0.25	14.97	< 0.001*
Head/neck vs. Chest	− 2.24	0.29	− 7.71	< 0.001
Udder/Scrotum vs. Chest	2.03	0.20	10.38	< 0.001*
Udder/Scrotum vs. Head/neck	4.27	0.29	14.61	< 0.001

*Statistically significant

The tick sequences generated in this study have been submitted to GenBank: *R. microplus* (acc. nrs. OR960937–OR960941), *R. turanicus* (OR960942), *R. annulatus* (acc. nrs. OR960943–OR960945), *R. decoloratus* (acc. nrs. OR960946 and OR960947), *R. geigyi* (acc. nrs. OR960948- OR960950), *R. evertsi evertsi* (acc. nrs. OR960951 and OR960952), *H. dromedarii* (acc. nrs. OR960953–OR960955), *A. variegatum* (acc. nrs. OR960956 and OR960957), *H. marginatum* (OR960958) and *H. rufipes* (OR960959).

## Discussion

### Effect of cattle characteristics on tick burden

Although nearly the same number of male and female animals were examined for tick infestation, the tick burden in male cattle was significantly higher than in females. This finding could be explained by reports that males are more often used for farming activities and hence stay longer in the field and move over longer distances to graze exposing them over a greater period to ticks ( Opara and Ezeh 2011; Musa et al. 2014).

Tick burden was significantly higher in cattle older than 3 years. Low tick burden in younger livestock has been observed in other studies (Rehman et al. 2017), and may be due to the small surface area of younger animals, the more frequent grooming (Mooring et al. 2000), or the suggested innate and cell-mediated immunity that confers protection from ticks (Okello-Onen et al. 1999). This impact of innate immunity warrants further study and may have implications for future control strategies.

## Tick species

Similar to previous studies in Ghana, *A. variegatum* was the predominant species found infesting cattle in this study (Bell-Sakyi et al. 1996; Walker and Koney 1999). *Amblyomma variegatum* impairs livestock growth (Stachurski et al. 1993) and has been reported as the main vector of *Ehrlichia ruminantium* which causes heartwater disease in animals ( Stachurski 2000; Esemu et al. 2013). It also transmits *Rickettsia africae* which causes African tick bite fever in humans (Kelly et al. 1996; Tomassone et al. 2018). *Hyalomma* ticks were also identified from the study sites. These ticks transmit pathogens that negatively affect cattle production (Jongejan and Uilenberg 2004). It is important to note that all the *Hyalomma* species identified in this study have been implicated as reservoirs or potential reservoirs of CCHFV (Bakheit et al. 2012; Gargili et al. 2017), suggesting a risk to cattle owners and abattoir workers. This study reports the first molecular identification of *Hyalomma dromedarii* in Ghana. *Hyalomma dromedarii* is primarily a camel tick but has been reported to infest domestic animals such as cattle, goats and sheep at a reduced prevalence rate (Abdullah et al. 2018). *Hyalomma dromedarii* is reported to transmit *Theileria annulata*, the causative agent of cattle theileriosis (Mamman et al. 2021; Omer et al. 2021).

In this study, *Rhipicephalus evertsi evertsi* was collected from cattle which can be compared to a previous study in Ghana (Addo et al. 2023b). Furthermore, this study again reports the first molecular identification of *Rhipicephalus turanicus*. *Rhipicephalus turanicus* is closely related to *Rhipicephalus sanguineus* and can be found in the Palaearctic and Afrotropical regions, infesting domestic animals such as dogs, cattle and sheep (Walker et al. 2003). It has been reported in Nigeria (Lorusso et al. 2013) and Angola (Sili et al. 2021). *Rhipicephalus* (*Boophilus*) *microplus* were also identified in this study. This invasive species has been previously reported in countries such as Burkina Faso, Cameroon, Ghana, Mali, Nigeria and Togo (Adakal et al. 2013; Madder et al. 2012; Biguezoton et al. 2016; Muhanguzi et al. 2020; Addo et al. 2023b). It exhibits high resistance to acaricides and aids in the transmission of *Babesia* species which affects cattle production (Jonsson 2006; Rodrigues and Leite 2013) and zoonotic pathogens *R. africae* (Ehounoud et al. 2016; Mediannikov et al. 2012; Reye et al. 2012) and *Coxiella burnetii* (Diarra et al. 2017). Apart from its resistance to the majority of acaricides, *R. microplus* has a higher rate of reproduction and a shorter generation duration (Baffi et al. 2008; Madder et al. 2011). This allows it to quickly populate an area and displace other closely related *Boophilus* species (Adakal et al. 2013; Tønnesen et al. 2004). *Rhipicephalus decoloratus*, *R. annulatus* and *R. geigyi* were other *Boophilus* species identified in this study. These species have been reported in Ghana (Addo et al. 2023b; Ntiamoa-Baidu et al. 2004), Cameroon (Awa et al. 2015; Silatsa et al. 2019) and Nigeria (Lorusso et al. 2013). There is an increased risk of diverse tick infestation in Ghana due to the transboundary movement of herders and their livestock. This calls for regular surveillance efforts to provide useful information needed to formulate tick control measures.

It was observed in this study that tick species were significantly associated with cattle across the ecological zones. For instance, *H. rufipes* and *H. truncatum* were more likely to infest cattle from the Deciduous Forest and Transition zone, respectively. The same was seen for *A. variegatum* and *R. microplus* which were more likely to infest cattle in the Deciduous Forest and Coastal savannah ecological zones, respectively. This finding is consistent with studies that suggest that tick abundance is influenced by habitats and ecological zones (Okello-Onen et al. 1999). The results of this study can also be compared to a recent report in Ghana that indicated tick species distribution varies across three ecological zones (Coastal, Guinea and Sudan Savanna zones) (Nimo-Paintsil et al. 2022). For control measures to be effective, they have to take into account the unique tick species distribution in each ecological zone. This information will aid in the choice of a suitable acaricide to control the tick populations and reduce infestation in cattle.

## Tick infestation and preferred attachment site on cattle

*Amblyomma variegatum* was found mostly attached to the udder/scrotum of the cattle which is similar to studies in Burkina Faso and Cameroon (Stachurski 2000, 2006). Furthermore, *A. variegatum* often attaches to the hairless regions of a host where it is safe to feed (Huruma et al. 2015). The second most predominant species was *H. rufipes* which had a high preference for the anal region of the cattle sampled. This preference for the anal region could be due to the moist nature of the anal region which could prevent desiccation during feeding. The tail of the animal, which often covers the anal region, prevents direct sunlight from reaching the ticks during the feeding process and further conceals them from the sight of animal handlers who sometimes remove them. Thus, acquiring a blood meal from the anal region would prove safer and conducive for *H. rufipes*. It was further observed that *H. rufipes* infestation was significantly different among the sexes of the cattle examined.

The seasonal distribution of tick species was not recorded in this study due to the different collection periods across the study areas. Furthermore, the breed of cattle was not recorded to give a clear indication of which breeds are more susceptible to tick infestation. Future investigations should include seasonal tick collections as well as specific cattle breeds to provide more information for creating effective control measures.

## Conclusion

In this study, cattle sampled within the different ecological regions of Ghana were infested with ticks of medical and veterinary importance. Host animal factors including age and sex significantly influenced the tick burden. The predominant tick species *A. variegatum* and *H. rufipes* were found mostly attached to the udder/scrotum and anal region of cattle, respectively. These tick species are known to cause significant damage to cattle production suggesting the need for the continuous monitoring of tick populations as well as tick-borne diseases within the country. Finally, risk factor identification can help formulate effective control measures to protect both animals as well as humans. Effective chemical control strategies should take into consideration the resting and feeding preferences of ticks on cattle.

### Supplementary Information

Below is the link to the electronic supplementary material.

Supplementary file1 (DOCX 16 KB)

Supplementary file2 (DOCX 15 KB)

Supplementary file3 (DOCX 23 KB)

Supplementary file4 (DOCX 20 KB)

## Data Availability

The article and its additional files contain supporting information for the conclusions drawn in this work. Upon justifiable request, the raw datasets utilized and examined in this study can be made accessible.

## Declarations

### Copyright assignment statement

CAPT Letizia, LCDR Diclaro II, LCDR Harwood and Dr. Nimo-Paintsil are military service members or employees of the U.S. Government. This work was prepared as part of their official duties. Title 17 U.S.C. §105 provides that ‘Copyright protection under this title is not available for any work of the United States Government.’ Title 17 U.S.C. §101 defines a U.S. Government work as a work prepared by a military service member or employee of the U.S. Government as part of that person’s official duties.
