# Supplementary material for: Risk factors affecting the feeding site predilection of ticks on cattle in Ghana
Source: Exp Appl Acarol. 2024 Apr 5;92(4):835–50. doi: 10.1007/s10493-024-00906-7 (PMC11065925; doi:10.1007/s10493-024-00906-7)
Supplement: Supplementary file 1 — Supplementary file1 (DOCX 16 KB) [file 10493_2024_906_MOESM1_ESM.docx]

**Title:** Risk factors affecting the feeding site predilection of ticks on cattle in Ghana

**Journal name**: Experimental and Applied Acarology

Seth Offei Addo^1,2*^, Ronald Essah Bentil^1,2^, Mba-tihssommah Mosore^1,2^, Eric Behene^1,2^, Julian Adinkrah^1,2^, Janice Tagoe^1,2^, Clara Yeboah^1,2^, Bernice Olivia Ama Baako^3^, Dorcas Atibila^4^, Sandra Abankwa Kwarteng^5^, Kwaku Poku-Asante^4^, Ellis Owusu-Darbo^6^, Victor Asoala^3^, Daniel Lartei Mingle^7^, Edward Nyarko^7^, Anne T. Fox^2^, Andrew G. Letizia^8^, Joseph William Diclaro II^9^, Shirley Nimo-Paintsil^2^, James F. Harwood^10^, Samuel Kweku Dadzie^1*^.

^1^Parasitology Department, Noguchi Memorial Institute for Medical Research, College of Health Sciences, University of Ghana, Legon, Accra, Ghana.

^2^U.S. Naval Medical Research Unit EURAFCENT, Accra, Ghana.

^3^Navrongo Health Research Centre, Navrongo, Upper East Region, Ghana

^4^Entomology Unit, Department of Clinical Laboratory, Kintampo Health Research Centre, Kintampo, Ghana

^5^Department of Theoretical and Applied Biology, College of Science, Kwame Nkrumah University of Science and Technology, Kumasi, Ghana

^6^School of Public Health, College of Health Sciences, Kwame Nkrumah University of Science and Technology, Kumasi, Ghana

^7^Public Health Division, 37 Military Hospital, Ghana Armed Forces Medical Service

^8^Infectious Diseases Directorate, Naval Medical Research Center, Silver Spring, Maryland, USA.

^9^Navy Entomology Center for Excellence, Jacksonville, Florida, USA.

^10^U.S Naval Medical Research Unit EURAFCENT, Sigonella, Italy.

*Corresponding authors

1. Seth Offei Addo: [sethaddo40@gmail.com](mailto:sethaddo40@gmail.com)

2. Samuel Kweku Dadzie: [sdadzie@noguchi.ug.edu.gh](mailto:sdadzie@noguchi.ug.edu.gh)

S1Table: Prevalence and mean distribution of ticks sampled from livestock

|  | **Number of Animals sampled** | **Number of ticks collected** | **Percentage Tick Abundance** | **Average of Ticks** |
| --- | --- | --- | --- | --- |
|  | **N (%)** |  | **(%)** | **Mean (SD)** |
| **Type of Animal** |  |  |  |  |
| Cattle | 388 (97.0) | 2,187 | 99.1 | 5.6 (5.4) |
| Horse | 11 (2.8) | 19 | 0.9 | 1.7 (1.0) |
| Sheep | 1 (0.3) | 1 | 0.04 | 1 (0.0) |
| **Sex of Animal** |  |  |  |  |
| Male | 198 (49.5) | 1,235 | 56.0 | 6.1 (6.6) |
| Female | 202 (50.5) | 972 | 44.0 | 4.9 (3.6) |
| **Age (years)** |  |  |  |  |
| ≤3 | 138 (34.5) | 562 | 25.5 | 4.0 (3.6) |
| >3 | 262 (65.5) | 1,645 | 74.5 | 6.5 (6.1) |
| **Regions (Ecological zone)** |  |  |  |  |
| Greater Accra (Coastal savannah) | 80(20.0) | 413 | 18.7 | 5.2(4.3) |
| Ashanti region (Decidous Forest) | 80(20.0) | 768 | 34.8 | 9.6(8.1) |
| Bono East region (Transition Zone) | 80(20.0) | 407 | 18.4 | 5.1(4.4) |
| Northern region (Guinea savannah) | 80(20.0) | 327 | 14.8 | 4.1(3.2) |
| Upper East region (Guinea savannah) | 80(20.0) | 292 | 13.2 | 3.7(3.3) |
| **Total** | **400** | **2207** | 100 | 5.5(5.4) |
