# Supplementary material for: Risk factors affecting the feeding site predilection of ticks on cattle in Ghana
Source: Exp Appl Acarol. 2024 Apr 5;92(4):835–50. doi: 10.1007/s10493-024-00906-7 (PMC11065925; doi:10.1007/s10493-024-00906-7)
Supplement: Supplementary file 2 — Supplementary file2 (DOCX 15 KB) [file 10493_2024_906_MOESM2_ESM.docx]

**Title:** Risk factors affecting the feeding site predilection of ticks on cattle in Ghana

**Journal name**: Experimental and Applied Acarology

Seth Offei Addo^1,2*^, Ronald Essah Bentil^1,2^, Mba-tihssommah Mosore^1,2^, Eric Behene^1,2^, Julian Adinkrah^1,2^, Janice Tagoe^1,2^, Clara Yeboah^1,2^, Bernice Olivia Ama Baako^3^, Dorcas Atibila^4^, Sandra Abankwa Kwarteng^5^, Kwaku Poku-Asante^4^, Ellis Owusu-Darbo^6^, Victor Asoala^3^, Daniel Lartei Mingle^7^, Edward Nyarko^7^, Anne T. Fox^2^, Andrew G. Letizia^8^, Joseph William Diclaro II^9^, Shirley Nimo-Paintsil^2^, James F. Harwood^10^, Samuel Kweku Dadzie^1*^.

^1^Parasitology Department, Noguchi Memorial Institute for Medical Research, College of Health Sciences, University of Ghana, Legon, Accra, Ghana.

^2^U.S. Naval Medical Research Unit EURAFCENT, Accra, Ghana.

^3^Navrongo Health Research Centre, Navrongo, Upper East Region, Ghana

^4^Entomology Unit, Department of Clinical Laboratory, Kintampo Health Research Centre, Kintampo, Ghana

^5^Department of Theoretical and Applied Biology, College of Science, Kwame Nkrumah University of Science and Technology, Kumasi, Ghana

^6^School of Public Health, College of Health Sciences, Kwame Nkrumah University of Science and Technology, Kumasi, Ghana

^7^Public Health Division, 37 Military Hospital, Ghana Armed Forces Medical Service

^8^Infectious Diseases Directorate, Naval Medical Research Center, Silver Spring, Maryland, USA.

^9^Navy Entomology Center for Excellence, Jacksonville, Florida, USA.

^10^U.S Naval Medical Research Unit EURAFCENT, Sigonella, Italy.

*Corresponding authors

1. Seth Offei Addo: [sethaddo40@gmail.com](mailto:sethaddo40@gmail.com)

2. Samuel Kweku Dadzie: [sdadzie@noguchi.ug.edu.gh](mailto:sdadzie@noguchi.ug.edu.gh)

S2Table: Average number of ticks collected from each portion of sampled cattle on the preferred part of attachment

| **Body part** | **Mean** | **±SE** |
| --- | --- | --- |
| Udder/scrotum | 3 | 0.27 |
| Anal region | 1.7 | 0.14 |
| Abdomen | 0.38 | 0.1 |
| Chest | 0.47 | 0.08 |
| Head/neck | 0.05 | 0.03 |
| Leg/Thigh | 0.09 | 0.02 |

SE means standard error
