# Supplementary material for: Risk factors affecting the feeding site predilection of ticks on cattle in Ghana
Source: Exp Appl Acarol. 2024 Apr 5;92(4):835–50. doi: 10.1007/s10493-024-00906-7 (PMC11065925; doi:10.1007/s10493-024-00906-7)
Supplement: Supplementary file 3 — Supplementary file3 (DOCX 23 KB) [file 10493_2024_906_MOESM3_ESM.docx]

**Title:** Risk factors affecting the feeding site predilection of ticks on cattle in Ghana

**Journal name**: Experimental and Applied Acarology

Seth Offei Addo^1,2*^, Ronald Essah Bentil^1,2^, Mba-tihssommah Mosore^1,2^, Eric Behene^1,2^, Julian Adinkrah^1,2^, Janice Tagoe^1,2^, Clara Yeboah^1,2^, Bernice Olivia Ama Baako^3^, Dorcas Atibila^4^, Sandra Abankwa Kwarteng^5^, Kwaku Poku-Asante^4^, Ellis Owusu-Darbo^6^, Victor Asoala^3^, Daniel Lartei Mingle^7^, Edward Nyarko^7^, Anne T. Fox^2^, Andrew G. Letizia^8^, Joseph William Diclaro II^9^, Shirley Nimo-Paintsil^2^, James F. Harwood^10^, Samuel Kweku Dadzie^1*^.

^1^Parasitology Department, Noguchi Memorial Institute for Medical Research, College of Health Sciences, University of Ghana, Legon, Accra, Ghana.

^2^U.S. Naval Medical Research Unit EURAFCENT, Accra, Ghana.

^3^Navrongo Health Research Centre, Navrongo, Upper East Region, Ghana

^4^Entomology Unit, Department of Clinical Laboratory, Kintampo Health Research Centre, Kintampo, Ghana

^5^Department of Theoretical and Applied Biology, College of Science, Kwame Nkrumah University of Science and Technology, Kumasi, Ghana

^6^School of Public Health, College of Health Sciences, Kwame Nkrumah University of Science and Technology, Kumasi, Ghana

^7^Public Health Division, 37 Military Hospital, Ghana Armed Forces Medical Service

^8^Infectious Diseases Directorate, Naval Medical Research Center, Silver Spring, Maryland, USA.

^9^Navy Entomology Center for Excellence, Jacksonville, Florida, USA.

^10^U.S Naval Medical Research Unit EURAFCENT, Sigonella, Italy.

*Corresponding authors

1. Seth Offei Addo: [sethaddo40@gmail.com](mailto:sethaddo40@gmail.com)

2. Samuel Kweku Dadzie: [sdadzie@noguchi.ug.edu.gh](mailto:sdadzie@noguchi.ug.edu.gh)

S3Table: Tick species associated with cattle geographical location

| **Tick species** | **Geographical location** | **No. of cattle examined** | **No. of cattle infested n (%)** | **OR (95%CI)** | ***p-value*** |
| --- | --- | --- | --- | --- | --- |
| *A. variegatum* | Coastal Savannah | 69 | 26(37.9) | 0.8(0.5-1.4) | 0.297 |
|  | Deciduous Forest | 80 | 54 (67.5) | 3.8 (2.3-6.5) | <0.001 |
|  | Transition zone | 80 | 41 (51.3) | 1.6 (1.0-2.6) | 0.057 |
|  | Guinea Savannah | 159 | 42 (26.4) | 0.3 (0.2-0.5) | <0.001 |
| *H. rufipes* | Coastal Savannah | 69 | 21 (30.4) | 1.0 (0.6-1.7) | 0.91 |
|  | Deciduous Forest | 80 | 12 (15.0) | 0.3 (0.2-0.6) | 0.001 |
|  | Transition zone | 80 | 25(31.3) | 1.0 (0.6-1.7) | 0.944 |
|  | Guinea Savannah | 159 | 62(39.0) | 1.9 (1.2-2.9) | 0.004 |
| *H. truncatum* | Coastal Savannah | 69 | 8 (11.6) | 1.4 (0.6-3.1) | 0.471 |
|  | Deciduous Forest | 80 | 12 (15.0) | 2.1 (1.0-4.4) | 0.053 |
|  | Transition zone | 80 | 2 (2.5) | 0.2 (0.0-0.9) | 0.032 |
|  | Guinea Savannah | 159 | 14 (8.8) | 0.9 (0.4-1.8) | 0.789 |
| *H. dromedarii* | Coastal Savannah | 69 | 0(0.0) | 1 | - |
|  | Deciduous Forest | 80 | 0 (0.0) | 1 | - |
|  | Transition zone | 80 | 0 (0.0) | 1 | - |
|  | Guinea Savannah | 159 | 0 (0.0) | 1 | - |

OR is defined as odds ratio; OR and *p*-value were obtained using a univariate logistic regression

S3Table continued: Tick species associated with cattle geographical location

| **Tick species** | **Geographical location** | **No. of cattle examined** | **No. of cattle infested n (%)** | **OR (95%CI)** | ***p-value*** |
| --- | --- | --- | --- | --- | --- |
| *H. marginatum* | Coastal Savannah | 69 | 2(2.9) | 1 | - |
|  | Deciduous Forest | 80 | 0 (0.0) | 1 | - |
|  | Transition zone | 80 | 0 (0.0) | 1 | - |
|  | Guinea Savannah | 79 | 0 (0.0) | 1 | - |
| *R. annulatus* | Coastal Savannah | 69 | 0 (0.0) | 1 | - |
|  | Deciduous Forest | 80 | 0 (0.0) | 1 | - |
|  | Transition zone | 80 | 0 (0.0) | 1 | - |
|  | Guinea Savannah | 159 | 0 (0.0) | 1 | - |
| *R. decoloratus* | Coastal Savannah | 69 | 0 (0.0) | 1 | - |
|  | Deciduous Forest | 80 | 0 (0.0) | 1 | - |
|  | Transition zone | 80 | 0 (0.0) | 1 | - |
|  | Guinea Savannah | 159 | 0 (0.0) | 1 | - |
| *R. evertsi evertsi* | Coastal Savannah | 69 | 0 (0.0) | 1 | - |
|  | Deciduous Forest | 80 | 0 (0.0) | 1 | - |
|  | Transition zone | 80 | 0 (0.0) | 1 | - |
|  | Guinea Savannah | 159 | 10(6.3) | 1 | - |

OR is defined as odds ratio; OR and *p*-value were obtained using a univariate logistic regression

S3Table continued: Tick species associated with cattle geographical location

| **Tick species** | **Geographical location** | **No. of cattle examined** | **No. of cattle infested n (%)** | **OR (95%CI)** | ***p-value*** |
| --- | --- | --- | --- | --- | --- |
| *R. geigyi* | Coastal Savannah | 69 | 0 (0.0) | 1 | - |
|  | Deciduous Forest | 80 | 0 (0.0) | 1 | - |
|  | Transition zone | 80 | 0 (0.0) | 1 | - |
|  | Guinea Savannah | 159 | 7(4.0) | 1 | - |
| *R. microplus* | Coastal Savannah | 69 | 11(15.9) | 2.3(1.1-5.0) | 0.031 |
|  | Deciduous Forest | 80 | 2(2.5) | 0.2(0.05-0.9) | 0.037 |
|  | Transition zone | 80 | 12(15.0) | 2.2(1.0-4.6) | 0.04 |
|  | Guinea Savannah | 159 | 10 (6.3) | 0.5(0.3-1.2) | 0.122 |
| *R. turanicus* | Coastal Savannah | 69 | 1(1.5) | 4.7(0.3-75.7) | 0.278 |
|  | Deciduous Forest | 80 | 0 (0.0) | 1 | - |
|  | Transition zone | 80 | 0 (0.0) | 1 | - |
|  | Guinea Savannah | 159 | 1 (0.6) | 1.4(0.08-23.2) | 0.796 |

OR is defined as odds ratio; OR and *p*-value were obtained using a univariate logistic regression
