# Supplementary material for: Risk factors affecting the feeding site predilection of ticks on cattle in Ghana
Source: Exp Appl Acarol. 2024 Apr 5;92(4):835–50. doi: 10.1007/s10493-024-00906-7 (PMC11065925; doi:10.1007/s10493-024-00906-7)
Supplement: Supplementary file 4 — Supplementary file4 (DOCX 20 KB) [file 10493_2024_906_MOESM4_ESM.docx]

**Title:** Risk factors affecting the feeding site predilection of ticks on cattle in Ghana

**Journal name**: Experimental and Applied Acarology

Seth Offei Addo^1,2*^, Ronald Essah Bentil^1,2^, Mba-tihssommah Mosore^1,2^, Eric Behene^1,2^, Julian Adinkrah^1,2^, Janice Tagoe^1,2^, Clara Yeboah^1,2^, Bernice Olivia Ama Baako^3^, Dorcas Atibila^4^, Sandra Abankwa Kwarteng^5^, Kwaku Poku-Asante^4^, Ellis Owusu-Darbo^6^, Victor Asoala^3^, Daniel Lartei Mingle^7^, Edward Nyarko^7^, Anne T. Fox^2^, Andrew G. Letizia^8^, Joseph William Diclaro II^9^, Shirley Nimo-Paintsil^2^, James F. Harwood^10^, Samuel Kweku Dadzie^1*^.

^1^Parasitology Department, Noguchi Memorial Institute for Medical Research, College of Health Sciences, University of Ghana, Legon, Accra, Ghana.

^2^U.S. Naval Medical Research Unit EURAFCENT, Accra, Ghana.

^3^Navrongo Health Research Centre, Navrongo, Upper East Region, Ghana

^4^Entomology Unit, Department of Clinical Laboratory, Kintampo Health Research Centre, Kintampo, Ghana

^5^Department of Theoretical and Applied Biology, College of Science, Kwame Nkrumah University of Science and Technology, Kumasi, Ghana

^6^School of Public Health, College of Health Sciences, Kwame Nkrumah University of Science and Technology, Kumasi, Ghana

^7^Public Health Division, 37 Military Hospital, Ghana Armed Forces Medical Service

^8^Infectious Diseases Directorate, Naval Medical Research Center, Silver Spring, Maryland, USA.

^9^Navy Entomology Center for Excellence, Jacksonville, Florida, USA.

^10^U.S Naval Medical Research Unit EURAFCENT, Sigonella, Italy.

*Corresponding authors

1. Seth Offei Addo: [sethaddo40@gmail.com](mailto:sethaddo40@gmail.com)

2. Samuel Kweku Dadzie: [sdadzie@noguchi.ug.edu.gh](mailto:sdadzie@noguchi.ug.edu.gh)

S4Table: Effect of Cattle age, sex and geographical location on tick burden for various body

|  |  | **Udder/scrotum** | | **Abdomen** | | **Chest** | | **Leg/Thigh** | | **Anal** | | **Head/Neck** | |
| --- | --- | --- | --- | --- | --- | --- | --- | --- | --- | --- | --- | --- | --- |
|  | Characteristics | Mean difference | *p-value* | Mean difference | *p-value* | Mean difference | *p-value* | Mean difference | *p-value* | Mean difference | *p-value* | Mean difference | *p-value* |
| **^a^Age of Animal** | |  |  |  |  |  |  |  |  |  |  |  |  |
| ≤3years | >3years | -1.7 | 0.1983 | -3.0 | 0.6074 | 0.17 | 0.0455 | -0.02 | 0.8625 | -0.4 | 0.6083 | -0.05 | 0.9804 |
| **^a^Sex** |  |  |  |  |  |  |  |  |  |  |  |  |  |
| Male | Female | 3.0 | <0.001 | -0.4 | 0.3774 | 0.1 | 0.8696 | -0.1 | 0.8409 | -1.2 | <0.001 | -0.1 | 0.7271 |
| **^b^Geographical location** |  |  |  |  |  |  |  |  |  |  |  |  |  |
| Coastal Savannah | Deciduous Forest | -7.5 | <0.0001 | -1.2 | 0.0048 | 0.8 | <0.01 | 0.01 | 1.00000 | 3.5 | <0.0001 | 0.24 | 0.0128 |
|  | Transition zone | -2.8 | <0.0001 | 0.3 | 0.7453 | 0.6 | 0.0025 | 0.01 | 1.0000 | 2.2 | <0.0001 | 0.24 | 0.0128 |
|  | Guinea Savannah | -0.8 | 0.0363 | 0.2 | 1.0000 | 0.2 | 1.0000 | -0.18 | 0.0026 | 2.1 | <0.0001 | 0.22 | 0.0329 |
| Deciduous Forest | Transition zone | 4.7 | <0.001 | 1.3 | <0.0001 | -0.9 | 1.0000 | -0.19 | 1.0000 | -0.9 | <0.001 | -0.05 | 1.0000 |
|  | Guinea Savannah | 6.7 | <0.0001 | 1.2 | 0.0002 | -0.7 | <0.0001 | -0.2 | 0.0002 | -1.4 | <0.0001 | -0.025 | 1.0000 |

| Transition zone | Guinea Savannah | 2.1 | <0.0001 | -0.1 | 0.7381 | -0.4 | <0.0001 | -0.2 | 0.0002 | -0.14 | 0.2260 | -0.025 | 1.0000 |
| --- | --- | --- | --- | --- | --- | --- | --- | --- | --- | --- | --- | --- | --- |

^a^*p-value* was obtained using the Mann-Whitney test

^b^p-value was obtained by Dunn’s multiple-comparison test for stochastic dominance using the Bonferroni correction
